# Sustainable irrigation technologies: a water-energy-food (WEF) nexus perspective towards achieving more crop per drop per joule per hectare

**DOI:** 10.1088/1748-9326/ac7b39

**Published:** 2022-07-06

**Authors:** Cuthbert Taguta, Tinashe Lindel Dirwai, Aidan Senzanje, Alok Sikka, Tafadzwanashe Mabhaudhi

**Affiliations:** 1 Bioresources Engineering Programme, School of Engineering, University of KwaZulu-Natal, P. Bag X01, Pietermaritzburg 3209, South Africa; 2 Centre for Transformative Agricultural and Food Systems, School of Agricultural, Earth and Environmental Sciences, University of KwaZulu-Natal, P. Bag X01, Pietermaritzburg 3209, South Africa; 3 Department of Soil-, Crop-, and Climate Sciences, University of the Free State, Bloemfontein Campus, P.O. Box 339, Bloemfontein 9300, South Africa; 4 Varmac Consulting Engineers, Scottsville, Pietermaritzburg 3209, South Africa; 5 Centre for Water Resources Research, School of Engineering, University of KwaZulu-Natal, P. Bag X01, Pietermaritzburg 3209, South Africa; 6 International Water Management Institute (IWMI-Delhi), NASC Complex, DPS Marg, Pusa Opp Todapur, New Delhi 110 012, India; 7 International Water Management Institute (IWMI), Southern Africa Office, Pretoria, South Africa

**Keywords:** agricultural water management, integrated, irrigation modernization, resilience, silo, trade-offs, water-energy-food nexus

## Abstract

Sustainable agricultural intensification requires irrigation methods and strategies to minimize yield penalties while optimizing water, land and energy use efficiencies. We assessed, from a silo-based and integrated water-energy-food (WEF) nexus perspective, the performance of irrigation technologies in different agro-climatic regions. Secondary to this, we assessed the impact of adopting systematic approaches such as the WEF nexus on improving efficiency in irrigated agriculture through irrigation modernization. The evidence-based perspectives of silo-based performances individually considered the metrics of yield (Y), water use efficiency (WUE), and energy productivity (EP). The WEF nexus approach applied sustainability polygons to integrate the three metrics into a nexus index representing the holistic performance of the irrigation technologies. Silo-based performance in temperate regions suggests net gains for WUE (+1.10 kg m^−3^) and Y (+6.29 ton ha^−1^) when transitioning from furrow to sprinkler irrigation, with a net loss in EP (−3.82 ton MJ^−1^). There is potential for a net loss on EP (−3.33 ton MJ^−1^) when transitioning from furrow to drip system in temperate regions. The best performance of irrigation technologies in dry regions in water, energy and food silos was achieved by sprinkler, drip and furrow irrigation systems, respectively. Thus, appraising irrigation technologies from a silos perspective promotes individual silos, which renders an unsustainable picture of the performance of irrigation systems. The integrative WEF nexus approach successfully highlighted the trade-offs and synergies in the nexus of water, energy and food in irrigated agriculture. Drip irrigation led all irrigation technologies in WEF nexus performance in dry (21.44 unit^2^), tropical (23.98 unit^2^), and temperate regions (47.28 unit^2^). Overall, the irrigation modernization pathway to drip technology from either furrow or sprinkler systems improves irrigated agriculture’s WEF nexus performance in all three regions for more crop per drop per joule per hectare under climate change. This can promote inclusive and sustainable irrigation development within the planetary boundaries.

## 1. Introduction

The global sustainability trajectory is fraught with numerous challenges. For example, growing populations have pushed the sustainable bounds of agricultural practices to achieve food and nutritional security. To ensure a ‘*safe operating agricultural production space*’, food production systems are shifting from silo or linear approaches to nexus systems for sustainable agricultural intensification. Irrigated agriculture is often presented as a panacea to food insecurity. However, it consumes approximately 70% of global blue water, with water use varying regionally, with African and Arab countries consuming 87% and 90%, respectively (Campbell *et al* 2017). Agrarian economies account for more than 80% of freshwater withdrawals (Uhlenbrook *et al* 2022); hence, agricultural activities pose a significant threat to land use and land cover changes, freshwater use and biogeochemical flows (nitrogen and phosphorous cycles), planetary boundaries (PBs) (Rockström *et al* 2009b), which we hypothesize have a negative trickling effect to local scale agricultural systems. The PB concept defines the safe operating space or the environmental limits within which humans should operate (Steffen *et al* 2015). Rockström *et al* (2009a) defined PBs as threshold boundary levels with respect to Earth operating systems that should not be exceeded to avoid catastrophic natural resources damage. The destabilization of the PBs requires a careful approach to resource utilization as a mitigation measure to achieve Sustainable Development Goal (SDG) 6.

Water, a chief driver in the agricultural production chain, requires systematic and careful utilization to minimize the continuous degradation of biodiversity and ecosystems (UN 2018, Smith *et al* 2019, Uhlenbrook *et al* 2022). Hence sustainable water use (SDG 6.4) can be coupled with other production components such as energy use in pressurized irrigated agriculture to form a nexus that potentially yields sustainability through zero hunger (SDG 2), job creation (SDG 7) and climate-resilient growth (SDG 1) (UN 2016, UNESCO 2020). Water, energy and food (WEF) are inextricably linked in a water-energy-food (WEF) nexus, and they crucially drive human survival, economic growth, poverty reduction and social development (Beekma *et al* 2021). The WEF nexus approach posits that water, energy and food are inextricably linked such that actions in one sector influence the others, synergistically or adversely, at different levels and scales (Hoff 2011, FAO 2014, IRENA 2015). Contrarily, the silos approach is the business-as-usual conventional policy- and decision-making that prioritizes the security of individual disciplinary sector(s) (Hoff 2011, Hoff *et al* 2019). Adopting sectoral or siloed approaches to driving desired outcomes has the potential for maladaptation, exacerbating trade-offs and threatening sustainability.

Simple and linear models that advocate scaling of current models of agricultural production, for example, in irrigated agriculture, risk transferring problems from one sector to another (DeLonge and Basche 2017); hence, there is a need for systematic and transformative approaches to managing resources and involved practices (Das *et al* 2020, Naidoo *et al* 2021). These approaches include the WEF nexus, which manages the three interconnected resources sustainably by reducing trade-offs and building synergies across sectors (Leck *et al* 2015, Rasul and Sharma 2016, Hoff *et al* 2019). The WEF nexus approach resonates with assertions that irrigation as a tool cannot comprehensively define sustainability. However, it is one of the multivariate tools that can be used to promote water use efficiency (WUE), water productivity (WP), and stabilize household income in low-income countries (LICs) (Pérez-Blanco *et al* 2020). However, implementation of the WEF nexus approach is still plagued by a lack of practical solutions, especially at local scales (Wada *et al* 2016, Fabiani *et al* 2020b).

Natural and anthropogenic activities have exerted pressure on water, energy, food, and land resources; the demand for these resources is expected to increase by 6%–55%, 40%–50%, 60%–100%, and 10%–20% by 2050, respectively (Hoff 2011, Scheierling *et al* 2014, Fernández García *et al* 2018, Scheierling and Treguer 2018, Das *et al* 2020, Beekma *et al* 2021). The wide ranges in future water and food demands can be attributed to the uncertainty and complexity of such projections and their drivers, such as climate change and socio-economic dynamics. Despite this, these scenarios explore probable future spaces that can aid in robust decision- and policy-making for sustainable development and management of resources. To sustainably achieve climate-resilient and inclusive growth, irrigation has been touted to be the panacea to food insecurity (Hamidov and Helming 2020) because it produces about 40% of the world’s food using energy and 70% of global blue water on 17%–20% of global cultivated land (Rost *et al* 2008, Scheierling *et al* 2014, Mateos 2016, FAO 2018, Fernández García *et al* 2018, Scheierling and Treguer 2018, Sadeghi *et al* 2020, AL-agele *et al* 2021, Wang *et al* 2021). For context, surface, sprinkler, and drip/trickle irrigation systems globally claim 75%, 20% and 5% of irrigated land, respectively (Mateos 2016, Fernández García *et al* 2018, Scheierling and Treguer 2018). Despite the significant current and projected investments in irrigation expansion which amounts to $8 billion (Ringler 2017), irrigated agriculture has failed globally to achieve the intended goals, such as minimizing yield penalties and sustainable livelihoods, mostly because of linear and sectoral approaches that have failed to consider the situation’s complexity. Contrary to widely held perceptions, water alone is not the panacea to solving low productivity and improving food security, but it is part of the solution (Higginbottom *et al* 2021). Similarly, the possibilities of expanding the irrigated agriculture area are limited by finite and scarce water and land resources, while some studies reported that modernization to pressurized irrigation saves water but penalizes with additional energy consumption and costs (Rodríguez-Díaz *et al* 2011, Rodríguez-Díaz 2013, Fernández García *et al* 2018). Thus, technology in irrigation management is only part of the bigger solution.

Irrigation management has traditionally focused on water alone while assuming non-limiting energy and optimum crop husbandry (Zwart and Bastiaanssen 2004). For example, Zwart and Bastiaanssen (2004) and Molden *et al* (2010) provided the global status of water productivity for major crops. Similarly, Molden and Gates (1990), Burt *et al* (1997), Molden *et al* (1998), Molden *et al* (2001) and Bos *et al* (2005) presented descriptions, practical guidelines, and case studies for assessing irrigation systems’ water and yield centric performance. Separate discussions have been done on energy productivity (EP) and use efficiency in irrigated agriculture (Pimentel 2009, Topak *et al* 2009, Lorzadeh *et al* 2011, Vural and Efecan 2012, Yuan and Peng 2017). However, many of these discussions and reports treat the individual inputs and factors in separate silos of water, energy and food, leading to limited information, understanding and quantification of water for food, energy for water and energy for food interlinkages (Siddiqi and Wescoat 2013, ADB 2017, Das *et al* 2020). Consequently, this has painted an incomplete and unsustainable picture of irrigation performance from the farmer’s perspective, economy, and informing policy (Scheierling *et al* 2014, Fernández García *et al* 2018).

Discovering solutions at the WEF nexus requires moving past yield and water as sole measures of success in irrigated agriculture, and holistically considering co-benefits and trade-offs in different irrigation systems, particularly as they relate to environmental and equity outcomes (DeLonge and Basche 2017). For example, Thirtle *et al* (2001) stated that a 1% increase in agricultural yields translates into a 0.6%–1.2% of households attaining food security; thus, it is imperative to know the associated water and energy use. This is crucial for small-scale farmer-led irrigation, wherein farmers utilize traditional irrigation systems to sustain their livelihoods from agriculture (FAO 2011). To this end, FAO (2011) proposed an integrative approach to managing irrigation schemes wherein it was cautioned that water might be critical in agriculture. Still, other factors of production can be equally important or dominant. Opportunities for a comprehensive assessment of irrigation systems lie in integrated approaches such as the WEF nexus, which highlights and enhances understanding of the linkages between the three interdependent components. Tools for analyzing the WEF nexus include sustainability polygons which are critical for presenting, reporting, and communicating the WEF synergies and trade-offs in irrigated agriculture. This is exemplified in their application in the Diagnostic Tools for Investment (DTI) in Water for Agriculture and Energy by FAO (2021a). They are used to present indices and indicators for investment needs and potential as institutional and policy. Fabiani *et al* (2020a, 2020b) used sustainability polygons in a WEF nexus framework and approach to show how different fertilization strategies affect productivity, inputs efficiency, and profitability of wheat production at the farm level in Italy, Greece and the Czech Republic. In India, Hochman *et al* (2017) used sustainability polygons to investigate the sustainability of different climate-smart agriculture adaptations in smallholder rice, maize, and seed cotton farming, under rainfed and irrigated conditions. de Vito *et al* (2017) used sustainability polygons within a footprint index-based WEF nexus framework to assess the sustainability of irrigation practice in Italy. Mabhaudhi *et al* (2019) and Nhamo *et al* (2020a, 2020b) used sustainability polygons in their iWEF tool to visualize the sustainability of WEF nexus at regional (Southern Africa), national (South Africa) and municipal scale (local) in South Africa, respectively. Thus, sustainability polygons are simple and effective tools for analyzing and characterizing the interconnections in WEF systems.

**Figure 1.**
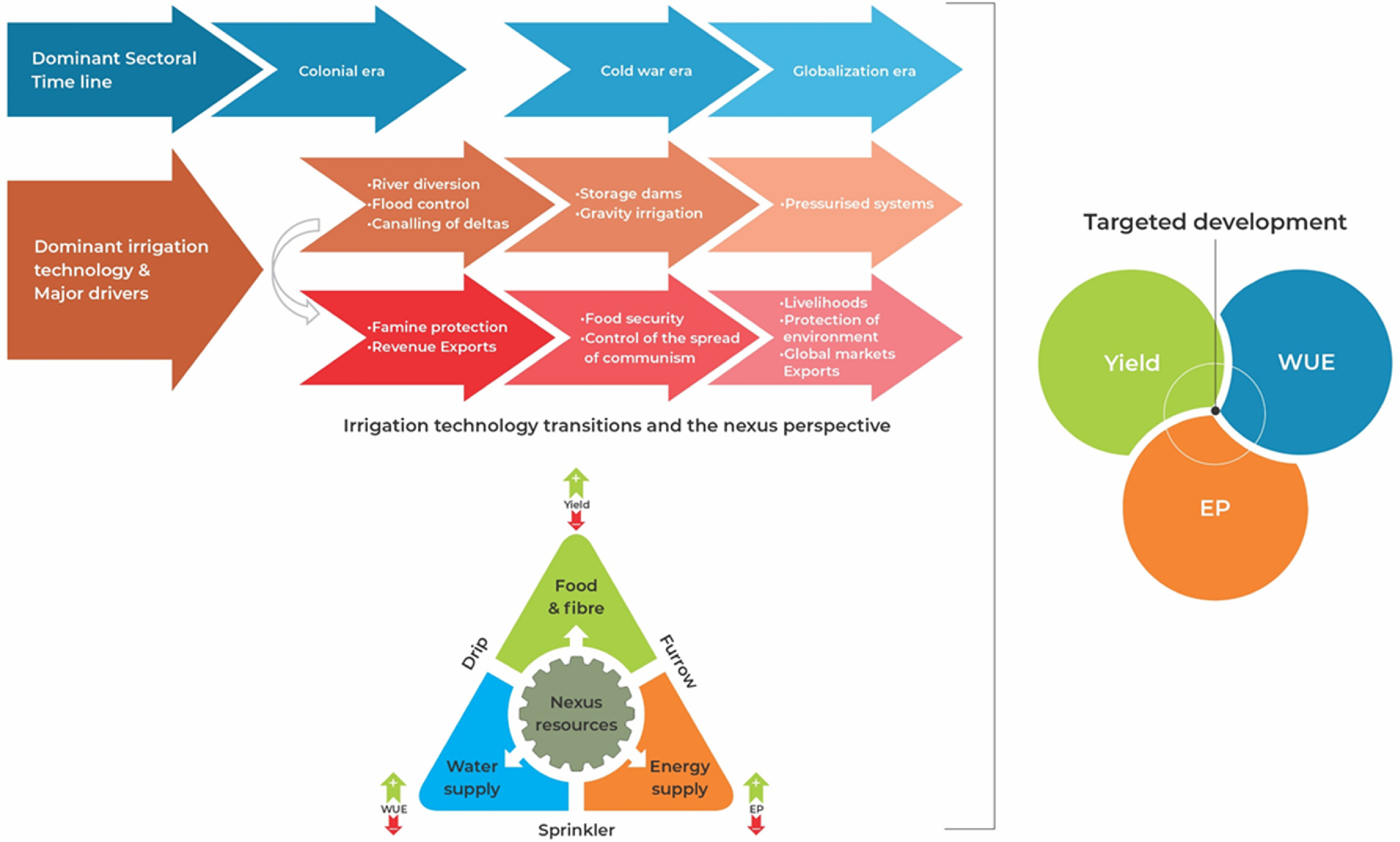
Conceptual framework.

To the best of our knowledge, there has not been an adequately integrated global assessment of the performance of different irrigation technologies during the modernization era (see figure 1) and their subsequent performances on a regional scale. It is unclear how a WEF nexus approach can systematically transform the design and implementation of irrigation technologies towards greater sustainability. To address this knowledge gap, this study sought to systematically review the available literature and potentially answer the following questions:

• How does the performance of irrigation systems from conventional ‘silo’ perspectives (considering silos independently) differ from a ‘nexus’ perspective (considering integrated WEF silos jointly)?
• Can a WEF nexus approach be applied to holistically appraise the performance of irrigation systems?
• What are the WEF nexus implications of irrigation modernization or transition from one system to another within a climate zone? Accordingly, what are the best irrigation modernization pathways for the different climate zones?

This review is intended to be a knowledge synthesis to guide and inform farmers, decision-makers, and practitioners on the benefits of adopting a WEF nexus approach to transform irrigation design and implementation towards sustainability, which we defined as transitions and designs that are conventionally favourable. An understanding of the trade-offs and synergies between farming systems optimization and efficient use of water and energy in irrigated agriculture would be needed to inform policy and investment decisions for enhancing productivity, viability and sustainability to address broader socio-economic development objectives (Giordano *et al* 2017, 2021). The WEF nexus addresses some multi-dimensional aspects of distributive and procedural justice. The WEF nexus is a social justice tool that addresses the processes and procedures involved in allocating natural resources and thus shaping the outcome. The global South is fraught with developmental challenges, and water and energy for food production are at the centre of the challenges. Nexus pathways are a potential solution for redressing societal imbalances through generating processes (procedural) for resource allocation and utilization and shaping the eventual livelihood outcomes (distributive).

## 2. Conceptual framework and methodology

### 2.1. Scenarios

Performances of irrigated agriculture have been investigated and reported on silos and indicators of water such as water productivity and WUE (Zwart and Bastiaanssen 2004); energy such as EP and energy use (Pimentel 2009, Topak *et al* 2009); and food such as yield. Previous integrated nexus case studies in irrigated agriculture include the water-energy nexus (ADB 2017) and the WEF nexus (Siddiqi and Wescoat 2013, Das *et al* 2020).

This study considered four approaches or frameworks reflecting different perspectives, in this case on irrigation performance:

(a) the WEF nexus framework as the integrated assessment approach considering all silos (energy, water, food) jointly;

while the three silo approaches only consider the individual silos:

(b) water silo represented by WUE, ignoring energy and food aspects,
(c) energy silo represented by EP, ignoring water and food aspects, and
(d) food silo represented by yield (or land use efficiency), ignoring water and energy aspects.

### 2.2. Definition of terms

The definition of WUE is a function of scale, data availability, and the practitioner, e.g. agronomist, irrigation scientist, or farmer. In this study, WUE (kg m^−3^) is defined as grain or utilizable yield harvested per unit volume of water used regardless of crop type, cultivar and growing season (Jat *et al* 2012, Varga *et al* 2013, Afzalinia and Ziaee 2014, Greaves and Wang 2017, Gunarathna *et al* 2018, Lokhande *et al* 2019, Zhang *et al* 2020):

\begin{align*}&amp;{\text{Water use efficiency}},\,{\text{ WUE }}\left( {{\text{kg}}\,{{\text{m}}^{ - 3}}} \right)\nonumber\\ &amp;\quad = {\text{ }}\frac{{{\text{Crop Yield }}\left( {\text{kg}\,{\text{h}}{{\text{a}}^{ - 1}}} \right)}}{{{\text{Water Consumption }}\left( {{{\text{m}}^3}\,{\text{h}}{{\text{a}}^{ - 1}}} \right)}}\end{align*}

For instance, an appropriate conversion method was employed to standardize the metrics when units were not given in kg m^−3^, e.g. WUE given in g litre^−1^ or kg mm^−1^. Similarly, the term ‘water productivity’ is sometimes used as a synonym for WUE with the same definition and units (Payero *et al* 2006, Djaman *et al* 2013). Grain yield (ton ha^−1^) was defined as grain yield yielded when the crop has reached maturity, whilst energy productivity EP (kg MJ^−1^) was defined as grain yield harvested per unit of energy supplied to drive the crop production process (Tabatabaeefar *et al* 2009). EP is determined as the ratio of crop yield to the energy input (Mohammadi *et al* 2008, Topak *et al* 2009, Mohammadi *et al* 2010, Mohammadi and Omid 2010, Banaeian *et al* 2020):

\begin{align*}&amp;{\text{Energy Productivity}},{\text{ EP }}\left( {{\text{kg}}\,{\text{M}}{{\text{J}}^{ - 1}}} \right) \nonumber\\ &amp;\quad= {\text{ }}\frac{{{\text{Crop Yield }}\left( {{\text{kg}}\,{\text{h}}{{\text{a}}^{ - 1}}} \right)}}{{{\text{Total Energy Input }}\left( {{\text{MJ}}\,{\text{h}}{{\text{a}}^{ - 1}}} \right)}}\end{align*}

The study focused on cereal crops, especially C4 crops (sugarcane, maize, sorghum, millet etc.), which are crops that utilize the phosphoenolpyruvate (PEP) enzyme to avoid photorespiration (Sage *et al* 1999).

### 2.3. Literature search

**Figure 2.**
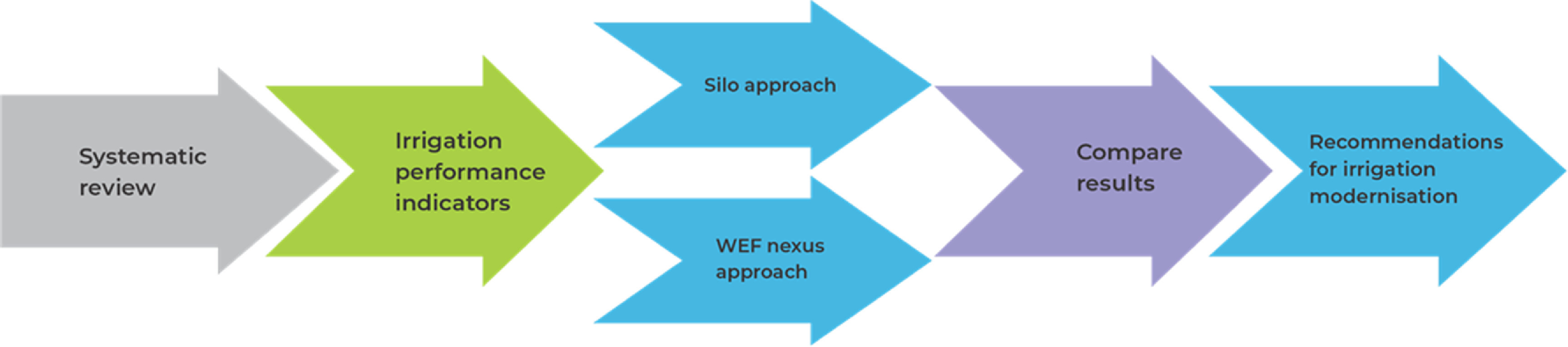
Study strategy.

A stepwise Preferred Reporting Items for Systematic Reviews and Meta-Analyses (PRISMA) protocol (Moher *et al* 2009) was applied in conducting a systematic review that informs the performance of different irrigation systems in different climate zones, from ‘silos’ and ‘WEF nexus’ approaches. The PRISMA protocol and guidelines assist in systematically searching, identifying, and selecting articles on search platforms and reviewing them through appraisal and synthesizing research evidence (Grant and Booth 2009). Page *et al* (2021a, 2021b) went on to update, further explain and elaborate PRISMA, which can also be adapted for other domains such as natural resources, agriculture, and management. At the same time, Fernandes Torres *et al* (2019) applied this framework in reviewing literature and proposing a systematic procedure for the nexus concept. For ease of understanding, the authors formulated a study pathway (figure 2) that presented scenarios, the target research group (scale), the corresponding indicators, and the measured outcome.

### 2.4. Literature handling

#### 2.4.1. Eligibility criteria

The study used the population, indicator, comparison, and outcome (PICO) strategy (table 1)_ENREF_11. The PICO strategy informed the search strategy and the subsequent inclusion–exclusion criteria. The study searched the following databases: Scopus, Web of Science (WoS) Core Collection, and Google Scholar (Date of the last search: 15 December 2021).

**Table 1.** The adapted PICO strategy used for literature handling.

	Criteria	Definitions
Population	Reviews and research articles focusing on selected geo-political locations.	Regions of concern were dry, tropical, and temperate climatic zones. The focus was on a global spatial scale.
Indicator	Research targeting irrigated agriculture that reported on WUE, EP, and yield.	The scales of operation were smallholder farmers and commercial farmers.
Comparison	Reported evidence on WUE, yield and EP for cereal crops under furrow, sprinkler, and drip irrigation systems.	All three irrigation systems were used for relative comparison.
Outcome	Dictates the desired measurable outcome, e.g. net positive gain or net negative gain (loss) obtained from irrigation technology transition.	The measurable output is obtained from transitioning from one irrigation technology/method to another.

#### 2.4.2. Search strategy and selection criteria

The search included intercontinental studies, all benchmarked on cereal crop production. Variables of interest were WUE, yield (Y), and EP. Boolean operators (**AND** and **OR**) were used, and typical search terms were: ‘irrigation*’ **AND** ‘crop production*’ or ‘WUE*’ **AND** ‘EP*’ **AND** ‘irrigation technology*’ **OR** ‘crop production*’. The crop term was subsequently replaced by cereal crops (maize, wheat, sorghum), and the irrigation term was also replaced by sprinkler/overhead, furrow, and subsurface/drip. The focus on these cereal crops was motivated by their strategic importance as major field and staple crops. The time period of publications was kept open. The authors used their discretion for literature screening and selection. Article screening was based on article title, abstract and locality. A two-step screening process (Dirwai *et al* 2021) was employed, firstly screening by title, and the second step involving screening by abstract and keywords. The adopted inclusion–exclusion criteria are summarized in table 2.

**Table 2.** Inclusion–exclusion criteria.

Inclusion	Exclusion
Article published in English	Articles not published in English
Original research in peer-reviewed journals	Articles from predatory journals
Conference proceedings	Full articles that could not be retrieved
MSc and PhD theses/dissertations Books Government gazettes	Articles with inadequate methodologies, insufficient results, and irrelevant discussion conclusions

The captured data from the retrieved records were entered into an Excel database. The retrieved sources lacked specific information on the type of water source from which the irrigation system was abstracting. The nature and type of water source likely impact the energy consumption and productivity of the irrigation system (Belaud *et al* 2020). Generally, the sources specified the quantitative and not qualitative aspects of the WEF silo components.

### 2.5. Geographical grouping of localities

The data collection did not restrict location to ensure global coverage and representation. The data were grouped into climate zones using the Koppen-Geiger climate classification by Kottek *et al* (2006). The study identified three dominant climate zones: arid or dry, equatorial or tropical, and warm temperate climates. To further refine the classification, we followed the Mathew *et al* (2017) and Mbava *et al* (2020) approach, which classified the several climate types using mean annual precipitation (MAP) and mean annual temperature (MAT). Tropical represents hot (MAT > 20 °C yr^−1^) and wet (MAP > 1000 mm yr^−1^) climate; subtropical depicts warm (MAT: 10 °C–30 °C yr^−1^) and arid to humid (MAP 100–1110 mm yr^−1^); temperate represents cool (MAT < 10 °C yr^−1^) and arid to moist (MAP: 120–1000 mm yr^−1^); while desert corresponds to warm (MAT > 15 °C yr^−1^) and dry (MAP: 0–100 mm yr^−1^). The environmental factors are summarized in table 3 below. In the absence of actual local characteristic climate data, the study used surrogate data from nearby locations (e.g. towns).

**Table 3.** Definition of environmental factors.

Environmental factor	Symbol	Units	Definition	Tropical	Sub-tropical	Temperate	Arid/desert
Mean annual precipitation	MAP	mm yr^−1^	Long-term (at least 30 year) mean precipitation per year for the study location from the papers.	>1000 (wet)	100–1110 (arid–humid)	120–1000 (arid–moist)	0–100 (dry)
Mean annual temperature	MAT	°C yr^−1^	Long-term (at least 30 year) mean temperature per year for the study location from the papers.	>20 (hot)	10–30 (warm)	<10 (cool)	>15 (warm)

### 2.6. Performance of irrigation systems

By systematically reviewing the retrieved literature, the performance of different irrigation systems was assessed by two approaches, i.e. silos and integrated WEF nexus perspectives.

#### 2.6.1. Statistical analyses (siloed approach)

A two-step multivariate analysis was carried out on the captured data. Firstly, two independent univariate analyses were carried out on (a) establishing a correlation amongst the variables irrigation technology, WUE, yield and EP, and (b) establishing a correlation amongst the variables: region/locality, WUE, yield and EP. Lastly, a multivariate analysis informed by the univariate methodologies was employed to establish an aggregated correlation amongst the variables: irrigation technology, EP, WUE, and yield. Before the multivariate analyses, the dataset was statistically tested for the assumptions, e.g. the normality test using the Shapiro-Wilks test and the equality test of variance–covariance amongst groups in which the groups are the irrigation technologies. An additional one-way ANOVA was employed to assess the statistical differences within groups.

#### 2.6.2. Integrated WEF nexus performance of irrigation systems by sustainability polygons

Irrigated agriculture is typical of the WEF nexus since it uses water and energy, among other inputs, to produce food and fibre (Hamidov and Helming 2020). Implementation of the WEF nexus approach is still lagging, possibly due to a lack of supporting evidence and tools that can fully capture it (Leck *et al* 2015, Liu *et al* 2017, Galaitsi *et al* 2018, McGrane *et al* 2019). Fortunately, simple tools such as sustainability polygons (spider-web diagram or radar chart) and integrated indices can be used to jointly compare multiple indicators across alternative options and scenarios, as well as systematically and quantitatively assess, evaluate, analyze, highlight, and visualize the synergies and trade-offs between them (Overton *et al* 2013, Flammini *et al* 2014, Colloff *et al* 2019, FAO 2021b). Sustainability polygons allow for an integrated graphic representation of multiple sustainability indicators through holistic visual summarization of sustainable competing alternative options (Hochman *et al* 2017).

The integrated assessment of irrigation systems from the WEF nexus perspective in the different irrigation system groups was conducted with sustainability polygons (FAO 2021b). Sustainability polygons holistically account for the interconnectedness of the three water, energy, and food indicators and determine the overall nexus performance (Flammini *et al* 2014). Sustainability polygons are applicable and useful even if the multiple options and scenarios have different indicators and metrics with different units of measurement, typical of water, energy and food indicators in irrigated agriculture (Overton *et al* 2013, Hochman *et al* 2017, Colloff *et al* 2019). In this graphic visualization tool, the indicator scores of each alternative (e.g. irrigation system) are joined with lines to form polygons whose relative area, enclosure or overlap is a nexus index that represents the sustainability or performance of the available options (Overton *et al* 2013, Hochman *et al* 2017, Colloff *et al* 2019). The option or alternative that completely encloses or has a larger polygon area is more sustainable.

In this study, the area enclosed by the sustainability polygon whose axes are the means of the WEF indicators (WUE, yield, EP) was used as a WEF nexus index (WEFNI) to measure the WEF nexus (see equation (3))

\begin{equation*}WEFNI = ASP\end{equation*}

where: $WEFNI$ is the WEF nexus index; $ASP$ is the area (in unit^2^) of sustainability polygon, which in this case is a combination of irregular triangles with known two sides and angle (120°) between them.

Local context priorities and value ranges in indicators dictate the need for weighting indicators, which is subjective and needs to be validated through stakeholder consultation (FAO 2021b), as exemplified in other studies by Mabhaudhi *et al* (2019), Nhamo *et al* (2020a, 2020b). However, to retain the original picture of irrigation performance, the indicators (WUE, yield, EP) were used without normalization and referencing or benchmarking, in contrast to approaches by other research work such as Hochman *et al* (2017) and Fabiani *et al* (2020a, 2020b). Similar to these mentioned studies, equal weights were assigned to each performance indicator to avoid bias, and since this study focuses on reviewing available literature without stakeholder engagement. An aggregated or overall WEF nexus performance or ‘WEF nexus profile’ (Fabiani *et al* 2020a, 2020b) for the different irrigation systems was produced by calculating the area enclosed by the sustainability polygon, similar to Frankowska *et al* (2019), who used ‘triangle graphs’ to visualize and quantify the impact of eight vegetables on the WEF nexus in the United Kingdom (U.K.).

### 2.7. Implications of irrigation modernization on water, energy and food

According to ADB (2017) and AL-agele *et al* (2021), pressure is growing on resources. The world must reduce the inputs used per unit of crop produced, for example, by installing efficient drip and sprinkler irrigation systems (FAO 2011). Hence the need arises to better understand the implications of adopting such technologies for various cropping systems and associated synergies and trade-offs (ADB 2017). In this study, the effects of such irrigation modernization are determined by considering transitions that are conventionally favourable or sustainable, i.e. in all the climate regions (Keller and Bliesner 1990):

(a) furrow to sprinkler, represented by *F* ⇒ *S*;
(b) furrow to drip, represented by *F* ⇒ *D*; and
(c) sprinkler to drip, represented by *S* ⇒ *D*.

The transition effects are quantified as changes in the magnitude of individual and integrated indicators for irrigation performance, with positive values being favourable (gain) and negative values being unfavourable (loss). To assess the implications of modernizing irrigation systems in irrigated agriculture, both the silos and the integrated WEF nexus perspectives were taken.

#### 2.7.1. Implications of irrigation modernization from a silos (water, energy and food) perspective

From a silo-based perspective, the gain (or loss) in irrigation technology performance in a modernization transition pathway is the difference in respective silo-based indicators for the two irrigation technologies in question, calculated as:

\begin{equation*}GSPT = S{P_f} - {\text{ }}S{P_i}\end{equation*}

where: $GSPT$ is the gain in silo-based performance as a result of the transition; $S{P_f}$ is the silo-based performance of the final state, i.e. target irrigation technology; $S{P_i}$ is the silo-based performance of the initial state, i.e. initial irrigation technology.

#### 2.7.2. Implications of irrigation modernization from an integrated WEF nexus perspective

From a WEF nexus perspective, the gain (or loss) in irrigation technology performance in a modernization transition pathway is the difference between the WEF nexus performances of the two irrigation technologies in question. For example, under any climatic region, the benefit or gain (unit^2^) of the transition from furrow to sprinkler irrigation systems is calculated as:

\begin{equation*}WEFNG = WEFN{I_f} - WEFN{I_i}\end{equation*}

where: $WEFNG$ is the gain in the WEF nexus index for an irrigation modernization transition pathway; $WEFN{I_f}$ and $WEFN{I_i}$ are the WEF nexus indices for the final and initial irrigation technologies, respectively. The WEF nexus index for an irrigation technology is calculated as the area enclosed by that irrigation technology’s sustainability polygon whose axes are the means of the WEF indicators (WUE, yield, EP), as explained in section 2.6.2 (equation (3)).

## 3. Results and discussion

**Figure 3.**
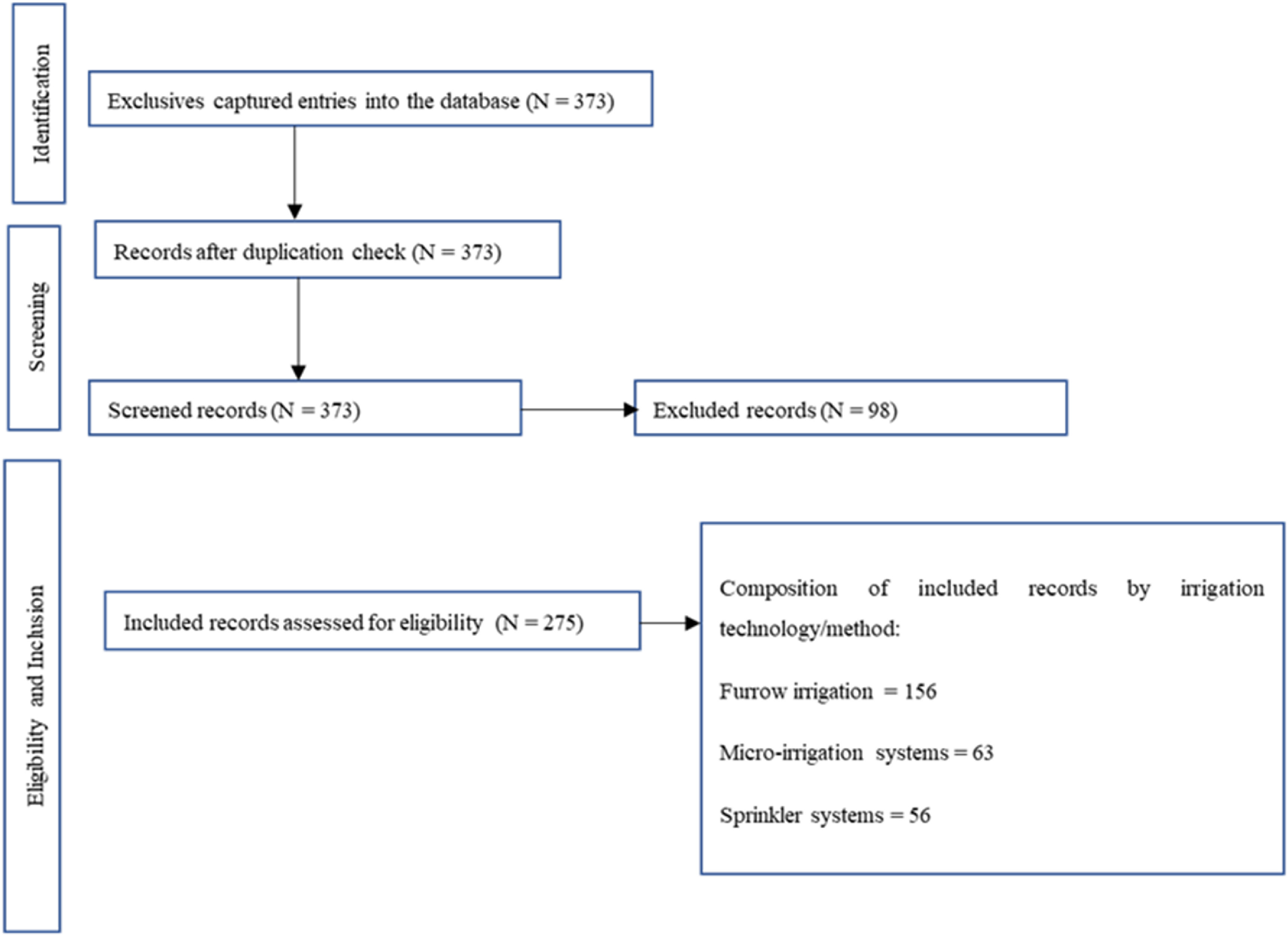
Systematic review flowchart of records based on PRISMA protocol.

Data charting was presented using a PRISMA flowchart (figure 3). The study utilized 275 articles (*N* = 275, 74%) out of the captured 373 articles across all three irrigation technologies. Furrow irrigation had the highest number of entries (*N* = 156, 57%), followed by drip irrigation systems (*N* = 63, 23%). Sprinkler entries (*N* = 56) constituted 20% of the database utilized entries across all the climatic regions. The three irrigation technologies were common across all the studied crops.

### 3.1. Irrigation systems performance through a silo perspective

#### 3.1.1. Global variations in WUE (water silo)

**Figure 4.**
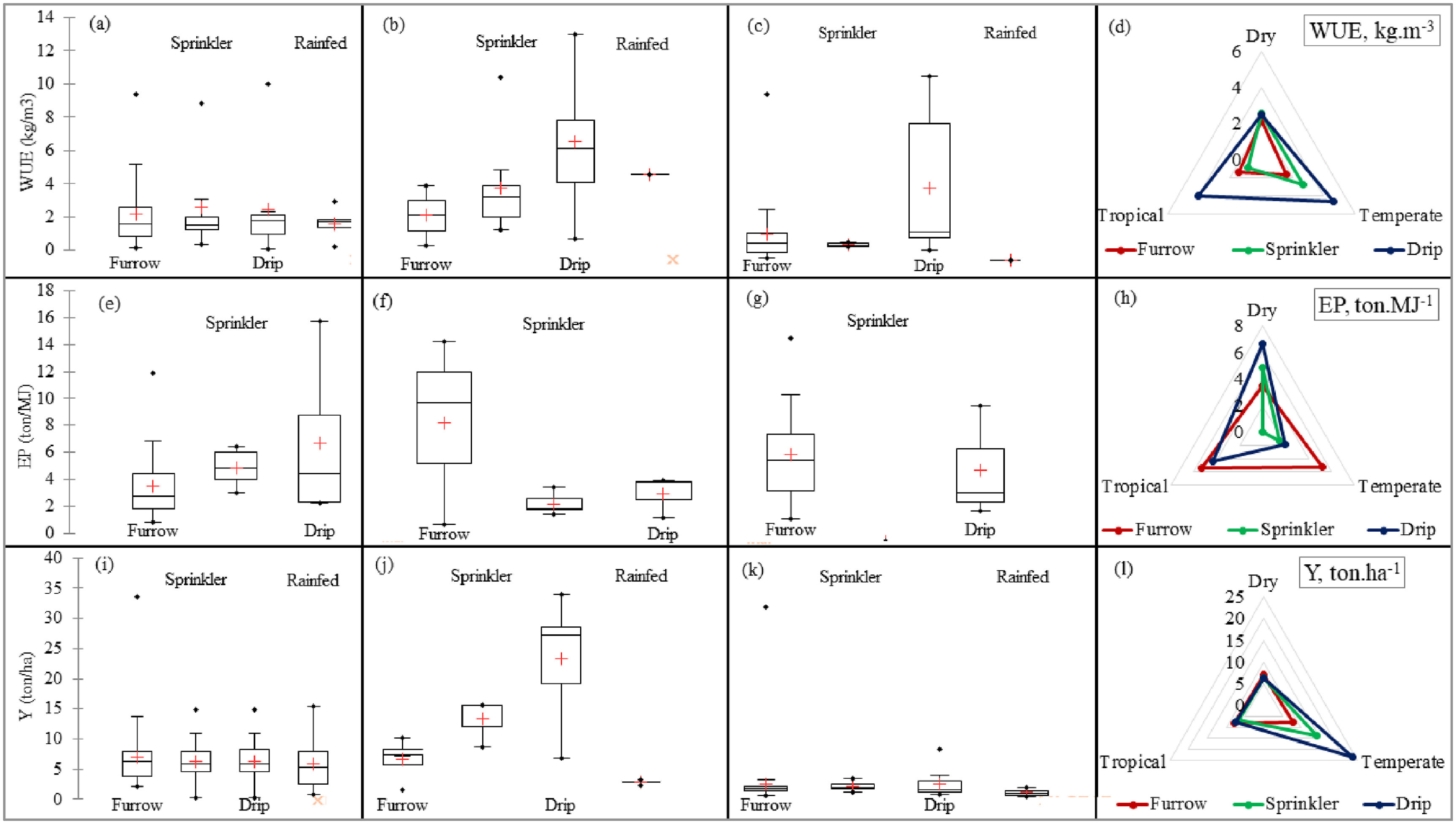
Water use efficiency (WUE) variation in (a) dry, (b) temperate, and (c) tropical climate, and (d) mean represented in sustainability polygon. Energy productivity (EP) variation in (e) dry, (f) temperate, and (g) tropical climate, and (h) mean represented in sustainability polygon. Yield (Y) variation in (i) dry, (j) temperate, and (k) tropical climate, and (l) mean represented in sustainability polygon.

The water silo-based performances of irrigation systems in different climate regions are shown in tables S.1, 4 and figures 4(a)–(d).

**Table 4.** Ordered silo-based and WEF nexus performance (best to intermediate to least) of irrigation systems.

Climate	Silo-based performance			Integrated performance, i.e., WEF nexus index
	Water (WUE)	Energy (EP)	Food (Y)	
Dry	*S, D, F*	*D, S, F*	*F, D, S*	*D, S, F*
Temperate	*D, S, F*	*F, D, S*	*D, S, F*	D, S*, F*
Tropical/Continental	*D, F, S*	*F, D, S*	*F, D, S*	*D, F, S*

WEF = water-energy-food, *D* = drip, *S* = sprinkler, *F* = furrow, * = almost similar/equal performance.

The mean WUE values for the three irrigation technologies in the dry climate zone were almost similar, with sprinkler irrigation recording a mean WUE (2.57 kg m^−3^), followed by drip irrigation (WUE = 2.47 kg m^−3^), and furrow systems (WUE = 2.17 kg m^−3^) (tables S.1, 4 and figure 4(a)). Cereal crops, especially C4 crops (sugarcane, maize, sorghum, millet etc.), which by definition are crops that utilize the PEP enzyme to avoid photorespiration (Sage *et al* 1999), exhibit a drought tolerance in dry or desert-like conditions, thus having a relatively high WUE (Mbava *et al* 2020). The WUE values were consistent with reported values of 2.3 kg m^−3^ in the arid regions of China (Deng *et al* 2006). For comparison and perspective, rainfed cereal production in the dry region had a mean WUE value of 1.55 kg m^−3^, slightly higher than the reported ranges of 0.5−1.01 kg m^−3^ under dryland farming (Xin and Wang 1998, Mu 1999, Deng *et al* 2006). The mean WUE values in the temperate climate zones were 1.62, 2.71, 4.63, and 3.30 kg m^−3^ for furrow irrigation, sprinkler irrigation, drip irrigation, and dryland scenarios, respectively (table S.1 and figure 4(b)). There were no statistically significant differences ($p\, &gt; {\text{ }}$ 0.05) in WUE values obtained in temperate climates across all three irrigation technologies. The data collected mainly involved furrow irrigated rice production in the temperate regions. The results revealed a statistically significant difference ($p$< 0.05) amongst the WUE means for the cereal grown under furrow, sprinkler, and drip irrigation, respectively, under continental climates (table S.1 and figure 4(c)). The reported mean WUE values were 0.85, 1.44, and 4.06 kg m^−3^ for sprinkler, furrow, and drip irrigation, respectively. The WUE values were inconsistent with the consensus that there tends to be an increase in WUE with a decrease in irrigated water (Mbava *et al* 2020). Climate also plays an important role in C4 production; thus, a low WUE for furrow and sprinkler irrigation signifies the un-adaptability of the crops to continental climates. C4 photosynthesis is optimal under tropical and sub-tropical conditions, where it minimizes yield penalties and improves WUE (Long and Spence 2013).

#### 3.1.2. Global variations in EP (energy silo)

The energy silo-based performances of irrigation systems in different climate regions are shown in tables S.1, 4 and figures 4(e)–(h), respectively.

The ANOVA revealed a non-statistical significance difference between irrigation technologies and EP in temperate regions. Pairwise comparison between furrow and sprinkler systems revealed a statistically significant difference (*p* < 0.05). A one-way ANOVA revealed a non-statistical significant EP difference between the sprinkler and drip systems. Drip irrigation recorded the highest mean EP (6.68 ton MJ^−1^), followed by sprinkler irrigation (EP = 4.86 ton MJ^−1^) and furrow systems (EP = 3.52 ton MJ^−1^) in the dry regions (tables S.1, 4 and figure 4(e)). This can be attributed to low and high energy input demands due to high and low irrigation water efficiencies in pressurized (drip and sprinkler) and furrow irrigation, respectively, to meet the high water demands by crops in dry regions. The mean EP values in the temperate climate zones were 1.42, 1.91, and 5.24 ton MJ^−1^ for sprinkler irrigation, drip irrigation, and furrow irrigation, respectively (table S.1 and figure 4(f)). This is consistent with Zhang *et al* (2021), who reported higher irrigation energy inputs and costs in drip irrigation than in furrow irrigation systems in China. There were no statistically significant differences ($p\, &gt; {\text{ }}$ 0.05) in EP values obtained in temperate climates and those obtained in dry climates across the three irrigation technologies.

Surprisingly, the results revealed a non-statistical significant difference amongst the EP means ($p$ > 0.05) for the cereal grown under furrow and drip irrigation, respectively, in continental or tropical regions (table S.1 and figure 4(g)). The reported mean EP values were 4.40 and 5.38 ton MJ^−1^ for drip, and furrow irrigation, respectively. It is worth mentioning that the reviewed literature did not yield any EP values under sprinkler irrigation in tropical regions.

#### 3.1.3. Global variations in yield (food silo)

The food silo-based performances of irrigation systems in different climate regions are shown in tables S.1, 4 and figures 4(i)–(l).

The Hotelling-Lawley test revealed a non-significant value ($p$ = 0.581) on the yield for the cereal crops grown under the three irrigation technologies and the dryland farming in the dry climate regions. The Hotelling-Lawley test also revealed a statistically significant difference ($p$ < 0.05) in yield recorded in the temperate regions. A one-way ANOVA revealed a statistically significant difference between the yields under furrow irrigation and those under drip irrigation (*p* < 0.05) in temperate climates, whilst non-significant statistical differences in yield were recorded between drip and sprinkler systems furrow and sprinkler irrigation.

The mean yield values for the three irrigation technologies in the dry climate zone was 6.64 ton ha^−1^, with furrow irrigation recording a mean yield of 7.07 ton ha^−1^, followed by drip irrigation (yield = 6.27 ton ha^−1^), and sprinkler irrigation systems (yield = 6.24 ton ha^−1^) (tables S.1, 4 and figure 4(i)). The yield values are slightly higher than the reported values of 1.70–5.0 ton ha^−1^ in the arid regions of West Asia and North Africa (Oweis *et al* 2000).

The mean yield values in the temperate climate zones were 7.76, 14.05, 23.56, and 3.30 ton ha^−1^ for furrow irrigation, sprinkler irrigation, drip irrigation, and dryland scenarios, respectively (table S.1 and figure 4(j)). There were statistically significant differences ($p\, &gt; {\text{ }}$ 0.05) in yield values obtained in temperate climates and those obtained in dry climates across the three irrigation technologies. The yield values recorded in the temperate regions were higher than those observed in the tropical regions. This is because irrigated crop production in tropical regions is characterized by pests and weeds that flourish in constantly moist and humid conditions (Rosenzweig and Liverman 1992). The conditions potentially impose yield penalties.

The results revealed a statistically non-significant difference ($p$ < 0.05) between the yields for the cereal grown under furrow and sprinkler and furrow irrigation and drip irrigation. However, a pairwise comparison (ANOVA) revealed a statistically significant difference between yields grown under drip irrigation and sprinkler irrigation ($p = 0.018$) in continental/tropical regions (table S.1 and figure 4(k)). The reported mean yield values were 6.67, 7.66, and 7.87 ton ha^−1^ for sprinkler, drip, and furrow irrigation, respectively. The possible explanation for the difference in yields between drip and sprinkler systems is that sprinkler irrigation promotes conditions for weeds and pests to flourish in humid and moist environments.

#### 3.1.4. Sustainability polygons of WUE, EP and yield

The overlaps of sustainability polygons in figures 4(d), (h) and (l) and the order of silo-based performances in table 4 show that trade-offs and synergies exist between WUE, EP and yield in cereal production across different irrigation systems in different agro-climatic regions. This is evidence of the prevalence of a nexus between water (WUE), energy (EP) and food (Y) in irrigated agriculture, hence the need for collectively and integratively considering these silo-based metrics when assessing the performance of irrigation systems because none of these individual silos can solely represent a comprehensive picture on performance. The sustainability polygons visualize the interconnections between water (WUE), energy (EP) and food (Y), thus visually summarizing the explanations presented in sections 3.1.1–3.1.3.

### 3.2. Interaction of WUE, yield, EP individual indicators with irrigation technology: a nexus approach

#### 3.2.1. WEF nexus performance of irrigation systems

**Figure 5.**
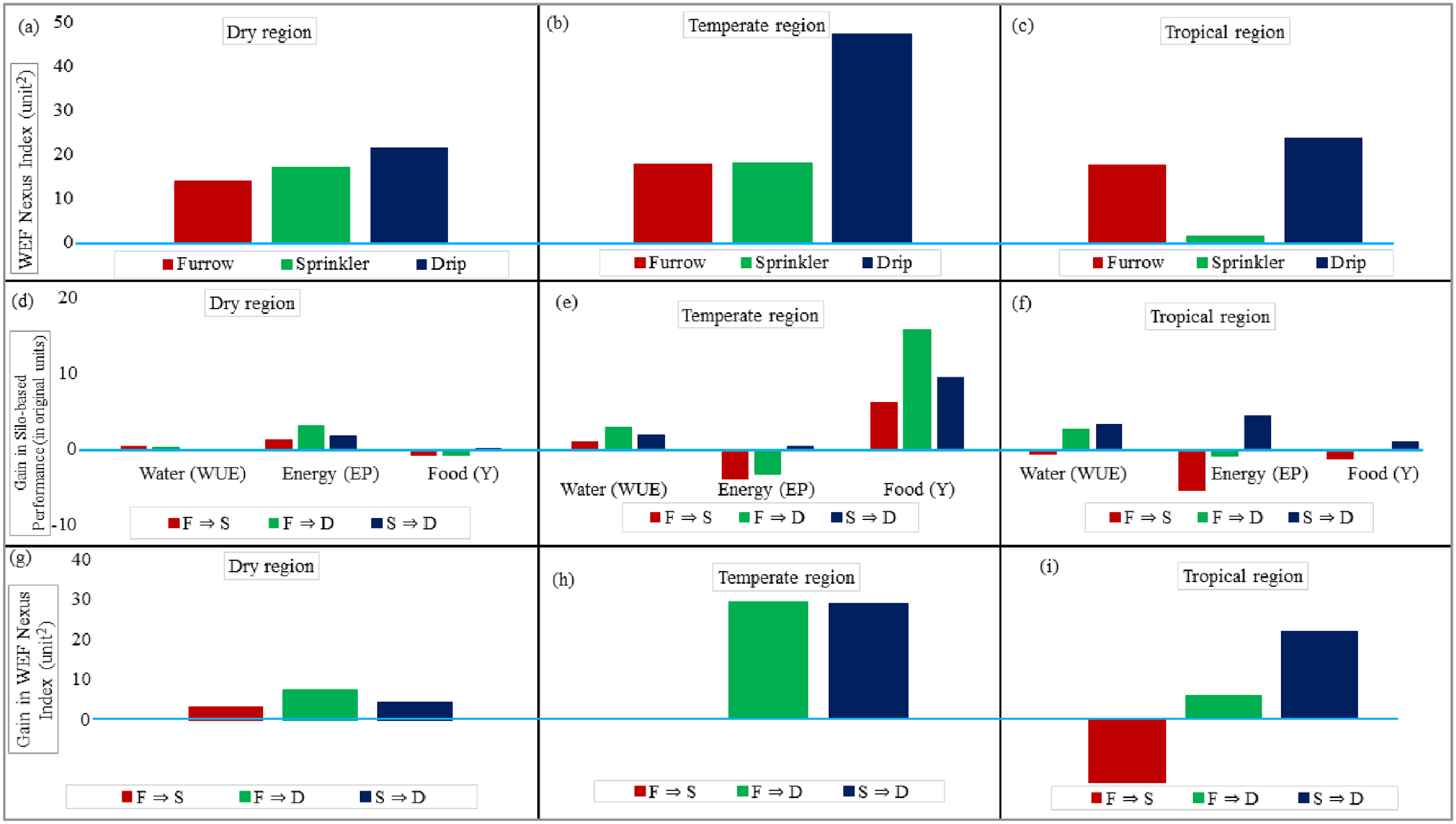
Integrated WEF nexus performance of irrigation systems in (a) dry, (b) temperate, and (c) tropical climates; impacts of irrigation modernization on the water, energy, and food performance from a silo approach in (d) dry, (e) temperate, and (f) tropical climates; and impacts of irrigation modernization on the WEF nexus in (g) dry, (h) temperate, and (i) tropical climates. (*The light blue line is at zero).*

The performances of irrigation systems from a WEF nexus perspective are graphically represented in figures 5(a)–(c) and table 4, wherein the WEF nexus performance was measured as an index represented by the area, in unit^2^, enclosed by the sustainability polygons integrating water (WUE), energy (EP) and food (Y) indicators as explained in section 2.6 (equation (3)).

In dry regions, drip irrigation systems had the highest WEF nexus performance (21.44 unit^2^), followed by sprinklers (17.12 unit^2^), and trailed by furrow systems (13.88 unit^2^) (figure 5(a)). In continental or tropical regions, drip irrigation systems have the highest WEF nexus performance (23.98 unit^2^), followed by furrow (17.83 unit^2^), and trailed by sprinkler systems (1.65 unit^2^) (figure 5(c)). The very low value of WEF nexus performance of the sprinkler irrigation systems in the continental or tropical regions can be attributed to the lack of data for EP in this agro-climatic region. In temperate regions, drip irrigation systems performed well in the WEF nexus (47.28 unit^2^), followed by sprinklers (17.99 unit^2^) and then by furrow systems (17.89 unit^2^), although the last two performed almost the same (figure 5(b)). Although drip irrigation leads in WEF nexus performance across all agro-climatic regions, the WEF nexus performance of sprinkler and furrow irrigation fluctuates, and care must be taken when selecting and recommending them. Potential transitions from one irrigation system to the other need to be quantified in WEF nexus terms to inform appropriate irrigation modernization pathways that maximize synergies, minimize trade-offs, and thus optimize the WEF nexus interactions.

### 3.3. Practical implications of irrigation modernization: a silo vs nexus perspective

#### 3.3.1. Implications of irrigation modernization from a silos (water, energy and food) perspective

The silo-based performances of irrigation modernization pathways are shown in figures 5(d)–(f), wherein the gain in performance was measured as the difference in respective silo-based indicators, i.e. water (WUE), energy (EP) and food (Y) indicators, as explained in section 2.6 (equation (4)). In dry regions, the transition from furrow to sprinkler and drip systems improved water use (+0.41 kg m^−3^) and energy use (+1.35 ton MJ^−1^) but compromised the cereal yields (−0.84 ton ha^−1^) (figure 5(d)). A change from sprinkler to drip led to a slight decrease and increase in water (−0.11 kg m^−3^) use efficiency and yields (+0.03 ton ha^−1^). This transition was accompanied by a significant improvement in energy productivity (+1.81 ton MJ^−1^).

In temperate regions (figure 5(e)), the move from furrow to either sprinkler or drip, respectively, saved water (+1.10 kg m^−3^ and +3.02 kg m^−3^) and improved yields (+6.29 and +15.81 ton ha^−1^) at the expense of energy usage (−3.82 ton MJ^−1^ and −3.33 ton MJ^−1^), with the drip system being more favourable. The sprinkler to drip transition improved WUE (+1.92 kg m^−3^), EU (+0.48 ton MJ^−1^) and yields (+9.51 ton ha^−1^). This transition failed to reject our hypothesis that sustainability can be achieved through the nexus approach. Adopting the nexus approach from bottom-up (local scale to regional) can potentially stabilize the PBs.

In tropical or continental regions (figure 5(f)), the transition from furrow to sprinklers resulted in reduced performances in water (−0.59 kg m^−3^) and energy use (−5.38 ton MJ^−1^) and decreased yields (−1.21 ton ha^−1^). Transitioning from furrow to drip system improved only water use efficiency (+2.62 kg m^−3^) but with trade-offs in the cost of efficiency in energy use (−0.98 ton MJ^−1^) and declined yields (−0.21 ton ha^−1^). Changing from sprinkler to drip led to improved productive water (+3.21 kg m^−3^) and energy use (+4.40 ton MJ^−1^), which subsequently translated to increased yields (+0.99 ton ha^−1^).

#### 3.3.2. Further discussion on silo-based approaches

Individual silo-based performance assessments exhibited an indiscernible trend (figure 4 and table 4). Silo performances revealed a non-consistent trend across the three silos (water, energy, and food) in the different climate zones. The irrigation systems performed differently across the three individual silos. From a sustainability point of view, one cannot assert that a particular irrigation system is the silver bullet for achieving water, energy, and food security in irrigated agriculture in the various regions. Thus, for sustainable resource use and to achieve SDG 6, a multiplicity of variables require attention and complement irrigation technology. To further highlight the compromised sustainability of siloed approach in achieving the interlinked SDGs 2, 6 and 7, there existed a high prevalence of trade-offs between water, energy, and food performances within a single irrigation system. The same chaotic results are evident in the irrigation modernization pathways, resulting in different performances of the same irrigation system regarding water, energy, and food. Selecting an irrigation system based on silo performance, such as yield or water, can penalize irrigation energy costs, such as furrow to drip in temperate regions (figures 5(d)–(f)), thus compromising sustainability at different scales. Thus, due to the apparent conflicting trade-offs, sacrifices, and penalties exchanged, selecting and recommending an appropriate irrigation system(s) in different climate zones from the results of silo approaches and individual performance metrics was difficult. This highlighted the need for approaches that integrate the different metrics into a measure of overall performance. According to Rodríguez-Díaz *et al* (2011) and Fernández García *et al* (2018), irrigation modernization processes undertaken in Spain, from open channel flow systems to pressurized networks, improved WUE by 21%. Still, system operation and maintenance costs quadrupled due to higher energy requirements (657%) for pumping pressurized systems compared to gravity-fed systems used previously. Hence a different approach is needed to integrate the different metrics from different silos into a measure of overall performance and produce integrated results that can be used for distinguished irrigation systems under different contexts such as climate.

As irrigation systems use energy to apply and distribute water to plants that produce the crop yield, considering water, energy, and food jointly in assessing the performance of irrigation systems leads to a better picture and characterization of the productive use of irrigation systems than considering the silos individually. Similar misrepresentations of WEF systems by silo-based frameworks were discovered in the Zambezi River Basin by Payet-Burin *et al* (2021), where silo-based frameworks under- or overestimated values on investments for irrigated agriculture expansion, hydropower capacity and thermal capacity by +22%, +7%, and −5% than the nexus framework, respectively.

#### 3.3.3. Implications of irrigation modernization from a WEF nexus perspective

The performances of irrigation modernization pathways from a WEF nexus perspective are graphically represented in figures 5(g)–(i), wherein the gain in WEF nexus performance was measured as the difference in WEF nexus indices represented areas enclosed by respective sustainability polygons, as explained in section 2.6 (equation (5)).

In dry regions (figure 5(g)), all transitions from furrow to sprinkler (+3.24 unit^2^), furrow to drip (+7.56 unit^2^), and sprinkler to drip (+4.32 unit^2^) had incremental gains in the WEF nexus performance of the system. However, the furrow to drip transition led to the highest net gain in performance, followed by sprinkler to drip, trailed by furrow to sprinkler. In the temperate regions (figure 5(h)), the transition from furrow to sprinkler irrigation is associated with a minimum and marginal increase (+0.10 unit^2^) in WEF nexus performance. Higher and almost similar improvements in WEF nexus performance are accrued by moving from furrow to drip (+29.39 unit^2^) and sprinkler to drip irrigation (+29.29 unit^2^). In tropical regions (figure 5(i)), the change from furrow to sprinkler amounts to a loss in the system’s WEF nexus performance (−16.18 unit^2^), probably due to increased energy consumption in pressurized irrigation systems. However, changing to drip from either furrow (+6.15 unit^2^) or sprinkler system (+22.34 unit^2^) accrued net gains in WEF nexus performance, with the latter transition being superior to the former.

#### 3.3.4. Further discussion on the WEF nexus approach

Unlike the unsustainable previous silo-based approaches (figure 4), the WEF nexus approach presented consistent and holistic performances of irrigation systems in different climate zones. Overall, drip irrigation systems optimized the WEF nexus across all the climatic regions because of precision methods associated with relatively higher yields and savings in water and energy. Sprinkler irrigation systems were second because they efficiently and productively used energy and water more than furrow irrigation systems (table 4). Thus, drip systems can be effectively and sustainably used in all climatic regions. In contrast, sprinkler systems are the second-best bet in dry regions, followed by surface systems being the least favourable option. Surface and sprinkler systems can be chosen with the same WEF nexus performance outcome in temperate regions.

It is worrisome that global statistics show that the irrigation systems with a higher WEF nexus performance, i.e. micro-irrigation are the least used (5% of agricultural land) than sprinkler (20% of agricultural land) and surface (75% of agricultural land) which have relatively lower WEF nexus performance (AL-agele *et al* 2021). This excludes the irrigators from LICs, e.g. farmer-led irrigation development (FLID), to participate in sustainable food production. This calls for gradual and careful irrigation modernization to drip irrigation, which shows promising results in efficient energy and water use to produce more food.

The WEF nexus approach to irrigation modernization showed that the dry region could benefit immensely from all transitions in this study, i.e. furrow to sprinkler, furrow to drip, and sprinkler to drip. All three irrigation transitions boost the WEF nexus performance in the temperate region, although the improvement is marginal in changing from furrow to sprinkler. Among the three irrigation transitions, furrow to sprinkler undermines irrigated agriculture’s overall WEF nexus performance in the tropical/continental regions. From a WEF nexus perspective, drip irrigation is the best endpoint of irrigation modernization in all climate regions.

### 3.4. A modernization shift: from silo to nexus perspective of irrigation performance

In this study, a single-factor assessment of irrigation systems yielded mixed results as the three factors or inputs alternately competed and outperformed each other. This highlights the insufficiency of this blinkered or narrow approach to irrigation system performance assessment and reinforces the need for an integrated WEF nexus approach that paints a relatively complete and comprehensive picture of irrigation performance. The thrust for improving and sustaining agricultural productivity and efficiency needs to move beyond the water and yield-centric ‘crop per drop’ approaches and counterproductive narratives (Scheierling and Treguer 2018) and embrace the holistic WEF nexus approach. The use of simple tools such as sustainability polygons and other WEF nexus analytic tools can be used to assess irrigation from a balanced and integrated perspective that optimizes productivity and sustainable use of input resources such as water and energy to produce yield. Although different from our case study approach and scale (transboundary basin), Payet-Burin *et al* (2021) applied the integrative nexus approach in planning WEF systems to save and optimize investments in irrigated agriculture expansion, hydropower capacity and thermal capacity in the Zambezi River Basin. de Vito *et al* (2019) used the WEF nexus approach to assess sustainable water resources management under irrigation in Italy. For a command canal irrigation system in India, the WEF nexus approach was successfully applied to optimize cropping patterns using context-specific conditions such as available water and energy resources (Das *et al* 2020). Irabien and Darton (2016) applied the WEF nexus approach to assess risks in greenhouse tomato production in Spain. However, when using sustainability and other related WEF nexus assessment techniques in local-scale case studies, there is a need to incorporate weights that express the relative importance of the three key sectors to provide the contextualized and realistic WEF nexus dynamics. The weights should be assigned from expert and stakeholder opinions depending on the prevailing priorities and WEF nexus dynamics of resource supply, demand, and scarcity. Such cross-sectoral collaboration and stakeholder engagement strengthen the effectiveness and validity of the WEF nexus approach. For example, some regions like Central Africa may have adequate water but scarce energy, thus requiring energy indicators to carry more weight than water indicators. Similarly, some regions have significant energy security but are water-stressed (e.g. South Africa, Middle East and North Africa), thus requiring water indicators to carry a higher weight in assessing the WEF nexus of irrigated agriculture.

Thus, in times when we are faced with insecurities of water, energy and food, as well as increasing energy costs, we must view the performance of irrigation systems in irrigated agriculture from an integrated WEF nexus to avoid compromises, sacrifices and minimize trade-offs (ADB 2017, López-Morales *et al* 2021). As irrigation systems use energy to apply and distribute water to plants that produce the crop yield, considering water, energy, and food jointly in assessing the performance of irrigation systems leads to a better picture and characterization of the productive use of irrigation systems than considering the silos individually. Farmers and practitioners can use this approach and resultant information to make informed choices of irrigation systems and be aware of the trade-offs between how much energy and water is expected to produce crop yield in particular climate zones. This is critical for the optimal choice of appropriate irrigation system for a particular crop in their region from a WEF perspective or the selection of non-optimal choice with awareness of the repercussions of such a choice concerning water use, energy use and expected yields. Thus, the irrigation sector should embrace the WEF nexus approach in the whole life cycle of irrigation systems and technology, from planning, design, operation, monitoring and evaluation. This approach can also be applied in ex-ante assessments, ex-post evaluations, economic, policy and environmental analysis, and planning and design that optimizes WEF nexus performance in irrigated agriculture. This can guide sustainable irrigated agriculture to feed the growing population without compromising water and energy security (Das *et al* 2020). Hence, the WEF nexus perspective in irrigated agriculture can inform us how to move towards more crop per drop per joule per hectare.

#### 3.4.1. Implications of the WEF findings on resource-poor communities

In many developing countries in the global South, sustainable irrigation development is not just seen as a means to increasing food production for food and nutrition security. It is located within a broader discourse of restorative justice, poverty, inequality and inclusion. Many rural resource-poor farmers have historically lacked access to water for irrigation due to water rights, tenure, and legacies of colonialism. This manifests as poor environmental injustice and poverty. As many governments are focusing on agriculture and irrigation development, in particular, to address socio-economic development among the resource-poor communities, the holistic and systematic WEF nexus approach, if adopted at higher levels, can improve livelihoods, build resilience, and amplify inclusion and beneficiation through mitigating trade-offs, maximizing synergies and enhancing sustainability (Mabhaudhi *et al* 2018, 2019). For example, WEF nexus informed irrigation transitions could facilitate improved agricultural water management (AWM) by large-scale farmers, translating to water-saving and potential increased water availability for new irrigators. This approach promotes distributive environmental justice through shared burdens and benefits across scales whereby financially able large-scale farmers’ transition to advanced irrigation technologies that improve AWM technologies for profits and improve environmental conservation for all. Access to water eliminates poverty; hence effectively and equitably utilizing water through scientifically-backed WEF-nexus irrigation technology transitions improves water adequacy and dependability across scales. Applying the WEF nexus approach in the development of resource-poor communities will potentially ensure the security of water, energy and food in such communities and support multiple sustainable development goals.

## 4. Limitations

Due to the choice and combinations of keywords used for the literature search in this study, some literature may not have been identified during the search. Still, this study provides a comprehensive representation of the work relevant to addressing the questions and objectives of our study. Our study focused on cereal crops because of their socio-economic importance and the wide range of irrigated crops. In this study, cereal crops (maize, wheat, sorghum) were reviewed in aggregate as a starting point to evaluate and demonstrate the wider applicability of the WEF nexus approach in irrigated agriculture. Water, energy and food are the default dimensions of the WEF nexus. However, other dimensions like cost, environment, climate change and ecosystem can expand the nexus and are outside the scope of this study. However, expanding the nexus increases the dimensionality, system dynamics, complexity, and uncertainty. Although the weights of individual WEF silos differ with the context in case studies, assigning them equally in our study was sufficient considering the nature of the current work (systematic review) in a global context. We appreciate that energy consumption and hence EP depends on the type of water source, although this information was missing in the studied sources.

## 5. Conclusion and recommendations

Sustainable agricultural technologies (SATs) are hinged on sustainable resource utilization to meet the global food demands. As such, irrigation is pivotal to producing more crops per drop of water per joule of energy per hectare of land used. Thus, irrigation practitioners need to be informed in selecting appropriate irrigation systems suitable for their conditions under prevailing land, water, and energy scarcity. The study concludes that:

(a) Sectoral approaches inhibit achieving the SDGs because of prevailing trade-offs and synergies among the water, energy and food metrics of performance. Furthermore, no irrigation system excels in all three silos in all agroclimatic regions, thus complicating the choice of appropriate irrigation systems and their modernization pathways in the climatic regions. Accordingly, each pathway leads to boosts in other silos at the expense of reduced performance in the other silo(s). These revelations showed that we need to move beyond sectoral approaches in WEF systems such as irrigated agriculture.
(b) Drip irrigation system was the champion of WEF nexus performance across all regions. Sprinkler and furrow come second in dry and tropical regions, respectively, while they have similar WEF nexus performance in temperate regions.
(c) In all irrigation modernization transition pathways and agro-climatic regions considered in this study, drip irrigation dominated in gain in WEF nexus performance. Transitions from furrow to sprinkler undermined the WEF nexus performance (−16.18 unit^2^) of the irrigated agriculture system in tropical regions due to increased energy consumption for pumping by sprinkler irrigation.
(d) Furrow to sprinkler irrigation transition in temperate regions brings about marginal WEF nexus gains (+0.10 unit^2^), thus requiring careful consideration of other factors such as costs when planning to go that route.

### 5.1. Resources valence and desired outcomes

The WEF-Nexus approach facilitates the valence of water, energy and food resources to generate sustainable agricultural outcomes and subsequently redress social inequities. To achieve this, potentially nexus-coherent technologies (e.g. drip irrigation) in tandem with proper government-led cross-sectoral basin-wide water management and control of water allocations procedures and processes should be adopted to avoid increased water use by land expansion, shifts to water-intensive crops, and groundwater over-exploitation. This would create a positive outcome valence from the input resources valence.

This study considered cereal crops (maize, wheat, sorghum) as a group, instead of focusing on them as individual crops, to demonstrate the wider applicability of the WEF nexus approach in irrigated agriculture without having to precisely target a particular crop. Thus, future studies should apply this approach to holistically assess the performance of irrigation systems in the production of individual crops or other groupings of crops, including vegetables and fruits, to expand the scope of focus on the precise application of the WEF nexus approach. Additionally, other transformative agriculture, irrigation and energy systems/technologies such as greenhouses, hydroponics, aquaponics and agrivoltaics that can potentially improve the WEF nexus need to be assessed from an integrated WE nexus perspective. For example, the agrivoltaics front will broaden the energy base, connect the unconnected and create a new value chain economic opportunities for resource communities while uplifting the standard of nutrition, health, and wellbeing). For example, agrivoltaics promotes the inclusion of smallholder farmers in energy generations and broadens their adaptation options. They can earn income from selling excess power when not irrigating or during drought. For rigorously applying a similar integrated nexus approach in localized case studies, we recommend contextualized weighting of the WEF sectors, probably by consulting experts, stakeholders, or relevant literature. This is critical to contextualize the local priorities and status of WEF resources.

Further studies should investigate the effect of water sources (e.g. surface, ground) on the WEF nexus performance of irrigation systems since the nature of water sources has implications on energy consumption and productivity. Other related WEF qualitative factors that need assessment include water quality, energy type (i.e. renewable or non-renewable), and crop types. Similarly, the irrigation modernization pathways recommended in this work should be assessed regarding other pertinent implications and advise farmers and practitioners accordingly. This would involve investigating the nexus of irrigation technologies with governance, environmental, economic, climate change, and socio-economic dimensions. Similar studies should also be conducted on various spatial and temporal scales, especially beyond the field, to better understand the synergies and trade-offs for real savings in resource use in irrigated agriculture to pursue more food and fibre. Another recommendation of note is upon data capturing; future studies should incorporate issues of terrain and different agronomic practices such as fertilizer use as they greatly impact the WUE variable.

## Data Availability

Data is publicly available on www.kaggle.com/datasets/tinashelindeldirwai/wefnexus.
